# Radiometric characteristics of some metallic ores and nonmetallic deposits: an example, Wadi Al-Allaqi, South Eastern Desert, Egypt

**DOI:** 10.1038/s41598-024-52912-9

**Published:** 2024-01-30

**Authors:** Mohamed Mostafa Gobashy, Mohamed A. El-Sadek, Mahmoud M. Mekkawi, Sultan Awad Sultan Araffa, Mohamed M. Ezz Eldin, Mohamed Hassan Khalil

**Affiliations:** 1 Geophysics Department, Faculty of Science, Cairo University, Giza, Egypt; 2 Nuclear Materials Authority, Maadi-Kattamia Road, P.O. Box 530, Cairo, Egypt; 3 National Research Institute of Astronomy and Geophysics (NRIAG), Helwan, Cairo, Egypt; 4 Egyptian Mineral Recourses Authority (EMRA), Cairo, Egypt

**Keywords:** Geophysics, Geology, Solid Earth sciences

## Abstract

Hydrothermal alteration processes are connected to many mineral formations, particularly auriferous deposits. In this study, airborne gamma-ray spectrometry (GRS) data and the analysis of radioactive materials (eU, eTh, and K) are applied to search for regions with hydrothermal alteration activity. An example is presented from Wadi Al-Allaqi, South Eastern Desert, Egypt. GRS was used to analyse various radiometric data to address potential mineral deposit areas, to map regions potentially showing metallic ore mineralisation, and to point out new geological mineral resources. The Kd (potassium deviation), “F” parameter and Th-normalisation of the K and eU anomalies were calculated, and locating new exploratory targets in the study area that exhibit high F-parameter, Kd, and K/eTh values was recommended. Additionally, the research region has a few isolated enriched spots of (K). Therefore, GRS data was used to characterise and estimate potential metallic ores, nonmetallic deposits, and gold ore zones associated with the alteration zones. Results show that most of the known mineral deposits and gold occurrences in the area, according to the metallogenic map of Egypt, are located in zones with a ratio value of (0.25–0.30) (K%/(U or Th ppm)) maps which may suggest a moderate degree of alteration. Also, most mineral deposits and gold occurrences are found in intermediate altered zones, or K-enriched sites, with a Kd% of (0.2. The work represents an attempt to map hydrothermal alteration zones associated with mineral deposits in the Wadi Al-Allaqi area. Generally, natural radiation characteristics and attributes suggest criteria that can be used globally for regional mineral exploration.

## Introduction

Gamma Ray Spectrometry (GRS) is a geophysical technique applied in various fields. It is utilised for geological mapping^1–6^, mineral exploration^6–8^, delineation of radioactive elements based on radiometric data^9^, and environmental radiation monitoring^6,10–13^. GRS data have also been employed for identifying contacts between different lithologic units^13–15^, distinguishing areas of hydrothermal alteration^16^, and establishing their relationship with base metal mineralisation processes. Additionally, GRS is used for gold and silver exploration in various geological environments^17^ because different rock types exhibit distinct concentrations of radioactive elements.

Using this method, the naturally occurring radioactive elements potassium (K), thorium (Th), and uranium (U) are measured at the outermost portion of the Earth's surface, where soil profiles and rock-forming minerals are present. Various types of volcanically associated massive-sulfide (VMS) base-metal and gold deposits are often associated with potassium alteration, typically in the form of sericite^18–21^.

The detection and mapping of potassium alteration associated with polymetallic volcanic-hosted massive sulphides (VMSs), magmatic-hydrothermal deposits (Au–Co–Cu–Bi–W–As), and porphyry Cu–Au–(Mo) deposits have been reported in numerous studies^22^, employing both aerial and ground gamma-ray spectrometry. Researchers have demonstrated that anomalously low eTh/K ratios, compared to typical lithological signatures, serve as a significant exploration signal and indicate potassium enrichment in specific geological contexts.

The naturally occurring radioactive elements in rocks are assessed using GRS to measure the concentrations of potassium, thorium, and uranium (K, eTh, and eU). The richness of uranium and thorium can be gauged during the decay of the parent elements by measuring the daughter nuclides formed and deducing the richness of the parent elements. The K% is measured directly, as gamma rays are released when 40K decays into argon. The concentrations of thorium and uranium are determined using the distinctive emission peaks associated with 208Tl and 214Bi^22^.

Airborne GRS data were collected over the southeastern desert of Egypt's Wadi Allaqi area (WAA) and its surroundings. This study focuses on assessing the distribution of radioactive minerals in local rock units, detecting hydrothermal alteration zones associated with metallic ores and nonmetallic deposits, and identifying new exploration targets by analysing radiometric properties, including eU, eTh, and K%. The constructed maps were utilised to investigate, identify, and concentrate on potential mineral resources, particularly gold, in WAA. Additionally, the study explores the global relevance of radiometric characteristics in mineral exploration.

### Geologic setting

**Figure 1:**
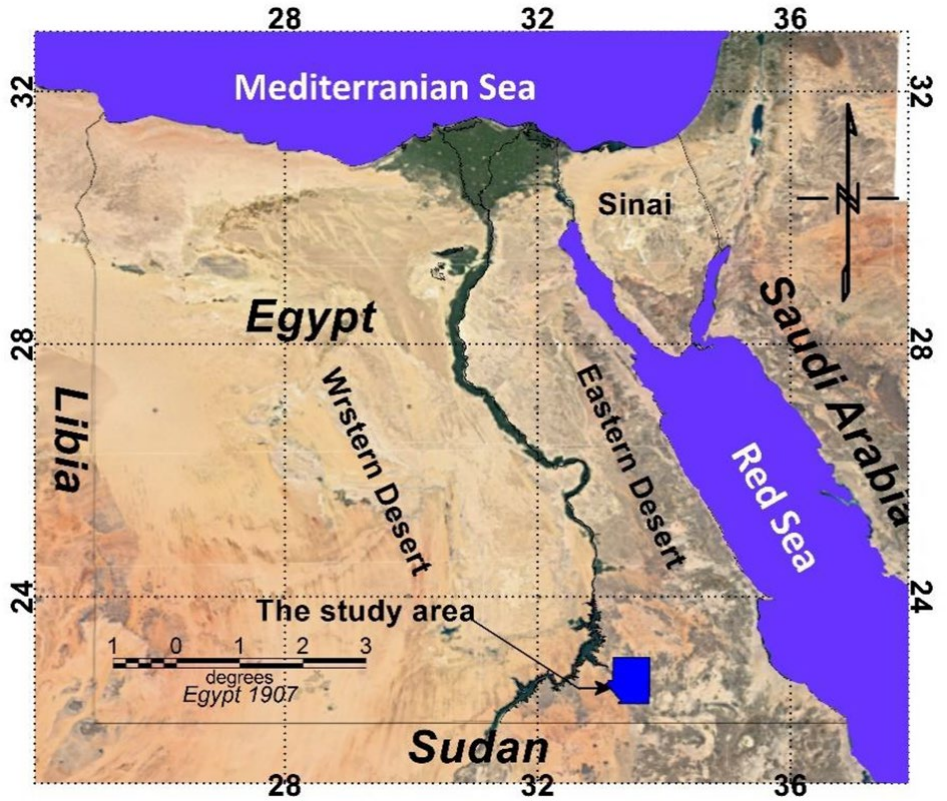
Location map of the study area (Modified Google image map).

The selected study area is positioned between longitudes 33° 07′ 30″ to 33° 45′ 15″ E and latitudes 22° 15′ 00″ to 23° 03′ 15″ N in the south of the Eastern Desert of Egypt, covering a surface area of 4800 km2 as illustrated in Fig. 1. This region encompasses various mineral deposits associated with hydrothermal processes, including talc, Au, Cu–Ni, U, and graphite deposits^23–27^.

Wadi Allaqi, the largest wadi in the South Eastern Desert, spans over 270 km on an NW–SE axis from the Red Sea Hills to its downstream confluence with the Nile Valley east of Lake Nasser. The Wadi Allaqi area features a diverse range of rock types, such as carbonatised ophiolites, island arc metavolcanic/volcaniclastic rocks, arc-related gabbro-diorite intrusions, syn-orogenic tonalite granodiorites, and late orogenic mozo/syenogranites.

**Figure 2:**
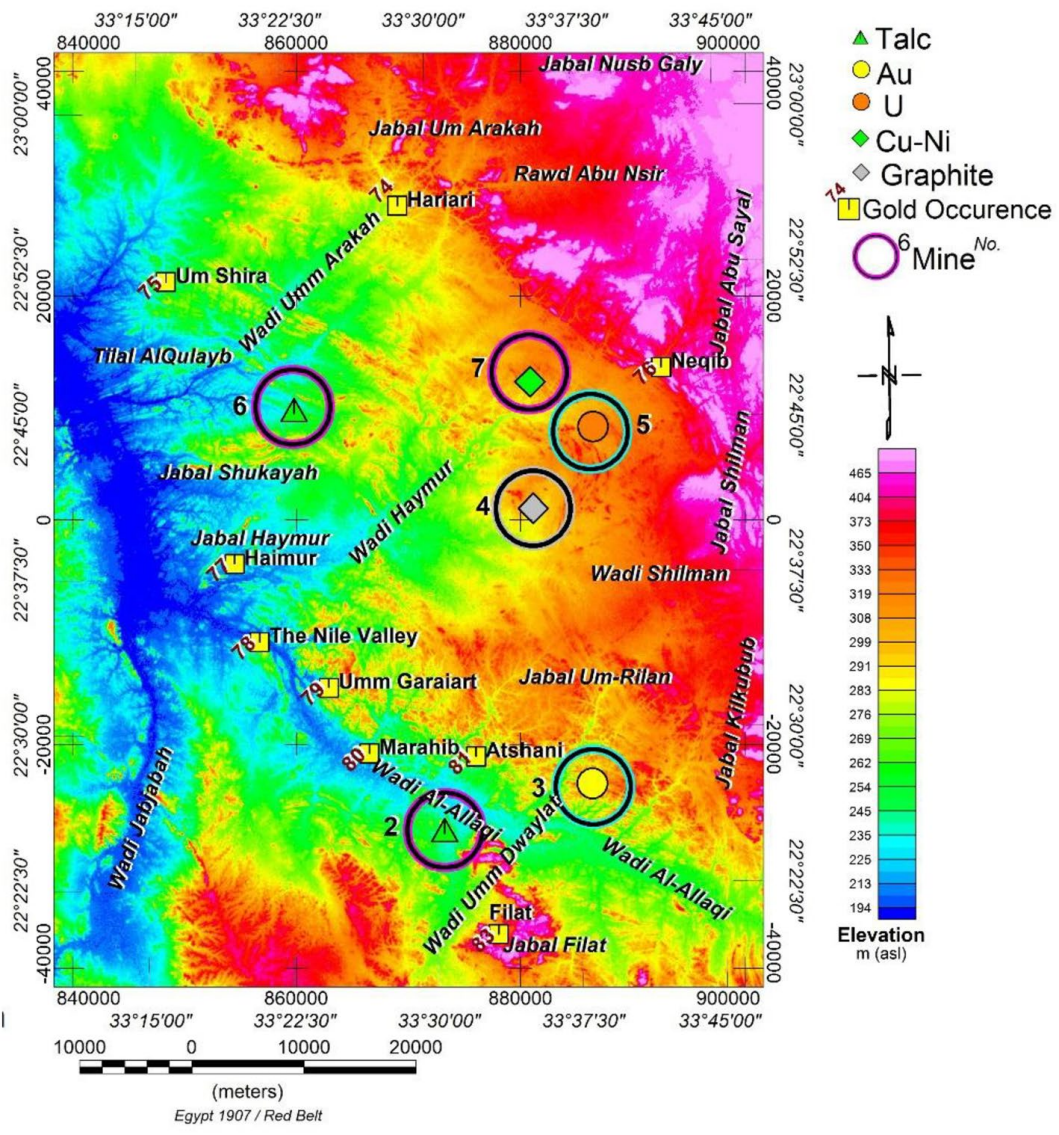
A 30 m resolution DEM of Area of Study (data downloaded from:http://wwwradar.jpl.nasa.gov/srtm/ (2013), visited 30 May 2023) (Yellow box) Gold deposits and occurrences in the study area in the south of the Eastern Desert of Egypt (compiled with permission from^28^. (Inside colored circle) Metallic ores and nonmetallic deposits (from^23^ with permission). The map is produced using Oasis montage V 8.4 software^29^.

Geomorphologically, the study region displays low, moderate, and high topographic features, as illustrated in Fig. 2. Outcrops exhibit sharp or gentle slopes with highly weathered surfaces. The landscape includes frequent occurrences of dunes, windblown areas, sand terrains, and playa deposits.

The predominant drainage pattern in the region follows a dendritic type, with upstream directed to the north. The wadis conform to major structural patterns. Notable wadis encompass Wadi Um Garayat (UG), Wadi Allaqi, Wadi Umm Arakah, Wadi Shilman, Wadi Jabjabah, and the prominent 250 m-tall UG Mountain. The area features low-relief topography, with 185–500 m above sea level elevations, easily accessible via the Aswan asphalt road.

**Figure 3:**
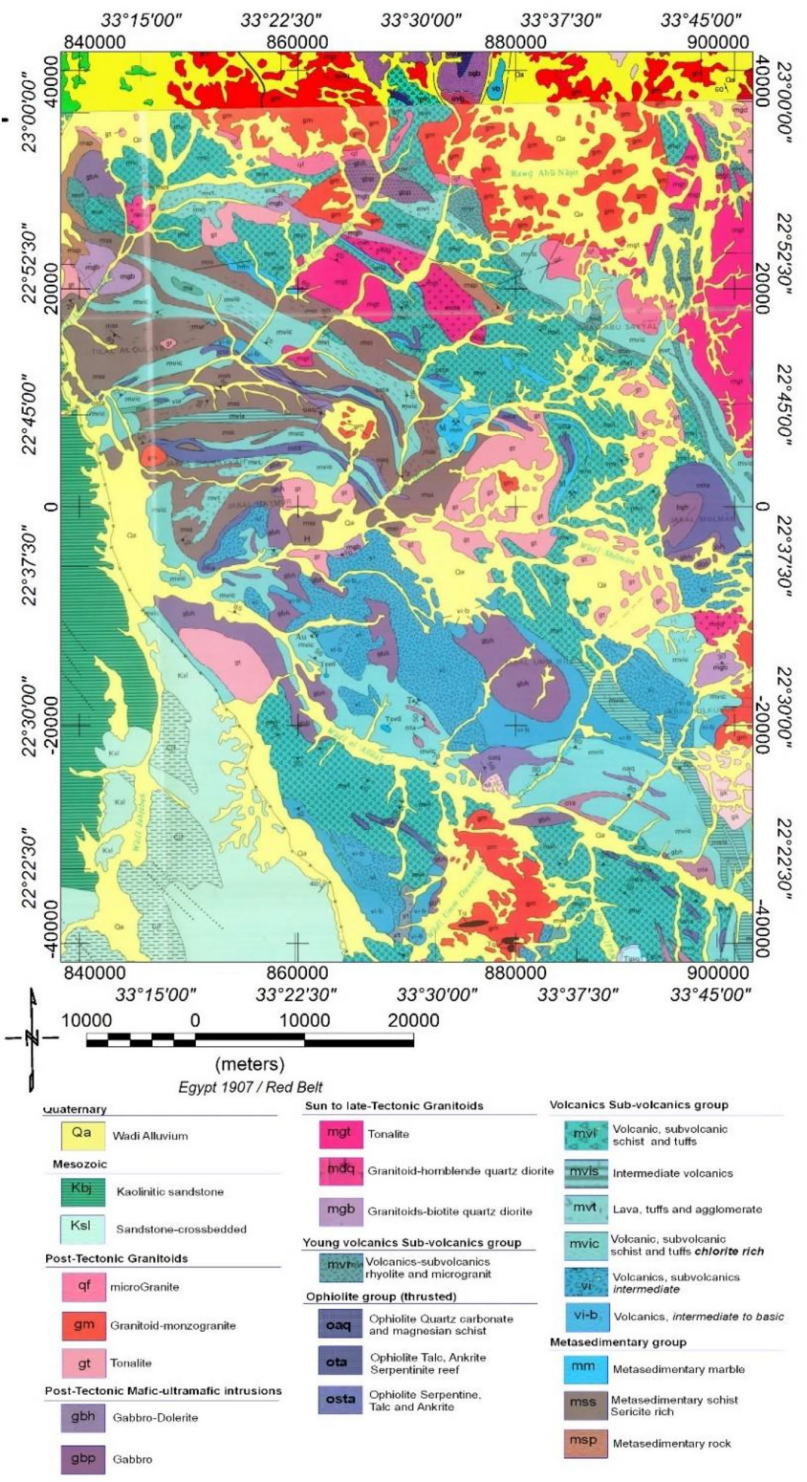
Geologic Map of Allaqi and its surrounding area, south Eastern Desert, Egypt. The map is modified with permission^32^ using Oasis montage V 8.4 software^29^.

The area under study encompasses rock units spanning a wide range on the geological time scale, from the Precambrian to the Quaternary, as depicted in Fig. 3. Most of this region is situated east of Aswan city, covering an area of about 4800 km2. The rocks in the vicinity of the Um Garayat mine are predominantly metavolcanic and metasedimentary^30^, displaying significant foliation in certain areas, along with intrusions of granodiorite-tonalite and meta gabbro-diorite (Fig. 3).

The metavolcanic rocks are made up of meta-andesite, meta-rhyolitic tuffs, meta-andesitic tuffs, and metadacite (Fig. 3). Metamudstone, Metasiltstone, and Metagreywacke with localised intercalations of marble, graphite schist, and Quartzite are less prevalent. The host rocks are broken in multiple directions by andesite, diorite, and trachyte dikes, as well as quartz veins, veinlets, and lenses. Sheared silty and graphite-bearing metasedimentary and tuffaceous rocks host gold-bearing quartz veins. The majority of veins dip 70° to the NE and strike N30°W (Fig. 3). Several mineralised veins span over one km and can reach a width of 1.8 m inside the areas of swelling^31–33^. Pervasive sulfidation (during metamorphism) is shown by chalcopyrite, covellite, chalcocite, and pyrite impregnations in the altered wall rocks next to the gold quartz veins^34^.

According to El-Kazzaz et al.^35^, the primary source of gold mineralisation in the southeast desert is a quartz vein system syn-tectonically deposited along the first deformation shear zone in granite. Veins and fractures formed in the readily fractured ductile shear zones, where auriferous metamorphic fluids were found. This process is linked to deformation and metamorphism rather than an epithermal origin. Zoheir et al.^36^ further revealed that the volcano-sedimentary sequence and ophiolites in the WA region had primarily metamorphosed in green-schist facies settings, even though peak metamorphic conditions were notably higher at depths up to 500–560 °C.

Peak metamorphism was documented, characterised by compressive deformation evident as sinistral shearing and regional folding^37^. The researchers concluded that improving the vectoring of promising zones would strengthen spectral and mineralogic features indicative of fluid concentration at structural connections. According to Sabet et al.^31^, areas in the WA region meeting the criteria for hydrothermal alteration along shear zones, particularly across the island arc of metavolcanic/meta-volcaniclastic rocks, are considered excellent candidates for drilling operations.

The region surrounding the Um Garayat (UG) metallic ore (Au) mine prominently features Neoproterozoic metavolcanic, metasedimentary, and metagabbro rocks. Examples of metasediments include graphite schist and meta-mudstone, with the graphite schist adorned by a stockwork of quartz veins and veinlets. The metavolcanic rocks encompass meta-andesites, meta-andesitic tuffs, meta-rhyolites, and meta-rhyolitic tuffs^31^. Alongside numerous quartz veins, veinlets, and lenses, andesitic, dioritic, and trachytic dykes have also intruded these rocks.

Several authors^32,34^ have examined the petrography of these rocks and concluded that intermediate to mafic basaltic andesite and dacite rocks are the predominant types in WA volcanics. El-Nisr^38^ asserts that these volcanic processes have been influenced by the continental arc/margin setting, low-grade metamorphism of the greenschist facies, and severe hydrothermal alterations superimposed on them. These rock processes span from calc-alkaline to low K-tholeiite. The study area encompasses rock units from the Precambrian to the Quaternary, as illustrated in Fig. 3. Table 1 summarises the rock units, their lithology, and their corresponding ages.

**Table 1:** Rock units, lithology, and the corresponding ages in the WA area.

Age	Rock units	Lithology
*Late proterozoic*	Meta-sedimentary rocks group	sericite biotite gneissic rocks (msp), sericite-rich schist (mss), talc, ankerite, and marble (mm)
	Calc alkaline Volcanics and sub-volcanics group,	Intermediate to base volcanic rocks (VI-B), intermediate sub-volcanic chlorite-rich rocks (VI), intermediate sub-volcanic schist and tuffs rich in chlorite (MVIC), intermediate lava, tuffs, and agglomerate rocks (MVT), intermediate volcanic rocks rich in sericite (MVIS), undifferentiated intermediate volcanic sub-volcanic schist, and tuffs (MVI)
	Ophiolite nappe /mélange group	Serpentine and talc (osta) at Jabal Shillman, Talc and ankerite (ota) at Wadi Haymur Ankerite, Quartz carbonate, and magnesian schist (oaq)
	The young calc-alkaline volcanic sub-volcanic group includes	Rhyolite flows, tuffs and microgranite (mvr)
	Syn to late tectonic granitoid (Older granites)	Quartz diorite (mgb), (mdq), and tonalite (mgt)
	Post-tectonic mafic ultra-mafic intrusions	Gabbro (gbp), and Gabbro dolerite (gbh)
	Post-tectonic potassic calc-alkaline granitoid (Younger granites)	Tonalite (gt), at Umm Ashira, Wadi Murrah, Wadi Shilman Mozogranite (gm), at Jabal Arakah,Rod Abu Nsir, Jabal Filat and Microgranite at Jabal Abu Sayal (qf)
*Paleozoic*, Carboniferous,	Cross-bedded sandstone (Cjf),	
*Mesozoic*, Cretaceous,	Cross-bedded sandstone (Ksl), and kaolinitic sandstone (Kbj) at Wadi Jabjabah	
*Cenozoic-*, Tertiary,	altered sub-volcanic dacite with pyrite gold mineralisation (Tsvd), (Umm Qurayat)	
Quaternary	wadi alluvium (Qa)	

### Radiometric measurements

The three radioactive elements found most abundantly in nature are potassium (K), uranium (U), and thorium (Th). Many rocks contain a significant amount of potassium, often an indicator of alteration in various mineral occurrences. Uranium and thorium, being both mobile and relatively immobile elements, are typically present at trace levels. Airborne gamma-ray maps commonly depict variations in K, eU, and eTh geochemistry from 30 to 50 cm below the Earth's surface. The radioelements, in general, are susceptible to loss through weathering that affects this thin layer. Additionally, their composition may alter due to mineralisation. The K levels in altered rocks may increase (enriched) or decrease (depleted or leached), while eTh may increase or decrease due to changes from hydrothermal alteration.s

### Data collection

The Western Geophysical Company of America, USA, conducted an aerial geophysical survey in 1984^39^. The survey covered a vast area in the Eastern Desert of Egypt, including the WA. It comprised both aeromagnetic and aero-radiospectrometric surveys. The two surveys were conducted simultaneously at 1.0 km intervals along parallel flight lines oriented NE–SW. Additionally, perpendicular tie lines were flown at 10 km intervals in an NW–SE direction. At a nominal sensor altitude of 120 m terrain clearance, multichannel radiospectrometric and total-intensity magnetic measurements were taken at 93 m intervals. The primary sensor components of the Aero-Service CODAS/AGRS 3000F computer-based digital data acquisition system were a Varian V-85 proton precession magnetometer mounted in a tail-stinger configuration and a high-sensitivity 256-channel airborne gamma-ray spectrometer (50 l NaI "Tl" crystals)^39^.

### Data processing and interpretation

#### Radiometric elements (TC, eU, eTh, and K%) maps

**Figure 4:**
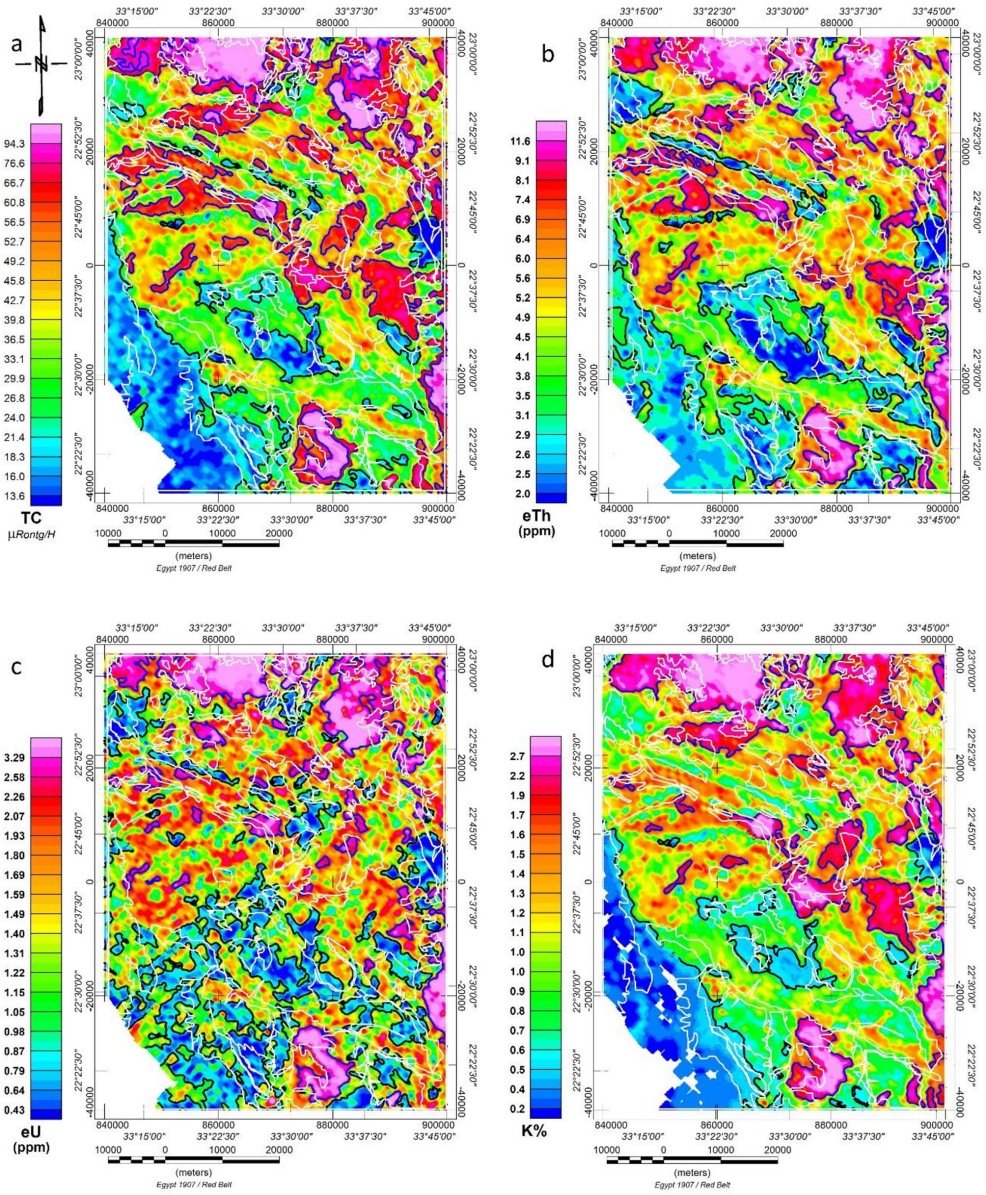
Radiometric elements of the study area. (**a**) Total count airborne gamma-ray, (**b**) Equivalent Thorium airborne gamma-ray, (**c**) Equivalent Uranium airborne gamma-ray spectrometric map of the study area., and (**d**) Potassium airborne gamma-ray spectrometric map. White lines represent the main geologic units, as shown in Fig. 4. All maps are extracted from the original survey^38^. Maps are produced using Oasis montage V 8.4 software^29^.

Gamma-ray spectrometer measurements were recorded as 1/10 of the measured values of micro-Rontgen/hour for the total count (TC), Uranium (eU), Thorium (eTh), and Potassium (K) concentrations. Therefore, dividing these measurements by (10) is recommended for corrections. A map is created for the total count and the three radiometric elements, as seen in Fig. 4a–d, to help visualise how those radiometric elements are distributed in space. The significant finding is a good agreement between the TC (Fig. 4a), K (Fig. 4d), eU (Fig. 4c), and eTh (Fig. 4b) maps and the geologic map after visualising, interpreting, and comparing thems.

According to its radiometric radiation level, the study area can generally be classified into three statistical levels: First, the lowest radiometric level in that zone (blue colour) is (lower than 2.3 μR/h for the total count, from less than 0.2–0.5% for potassium, less than 1.0 ppm equivalent for uranium, and from less than 2.0 ppm to 3.3 ppm equivalent for thorium). Sedimentary cover, ophiolite (serpentine, talc, and ankerite) (osta) rocks, mafic–ultramafic intrusions (gabbroic rocks) (gbh, gbp), and meta-sedimentary (marble, schist) (mm, mss, msp) rocks are all present in the research region along with this level.

The second level zone, ranging from yellow to bright red, contains values of 2.3–5.6 μR/h for total count, 0.5–1.65% for potassium, 1.0–2.3 ppm equivalent uranium, and 3.3–7.5 ppm equivalent thorium. This level is associated with some post-tectonic tonalite (gt) and a few small meta-volcanic sites in the research area.

The third level (red to violet colour) has a total count range of (5.6 to more than 15 μR/h), a potassium range of (1.65–3.5%), a uranium range of (2.3–10.5 ppm), and a thorium range of (7.5–20 ppm). In the study area, this level is accompanied by exposed granitoid rocks such as post-tectonic granitoid (younger granites), micro-granite, monzogranite, and tonalite (qf, gm, gt). Some tonalite units do not have high or moderate radioelement concentrations, and the third level does not characterise older granites.

### Ratio maps

Because some elements are stable, while others are easily transported or mobilised by air or hot (hydrothermal) solutions during certain tectonic events, structural movements, and alteration processes, various ratios of radiometric element maps were developed. Multiple methods have been proposed for detecting hydrothermally enriched or depleted zones and mineralisation zones^40–43^.

To mitigate the effects of the environment, lithology, leaching, and weathering on K and eU concentrations, the radioactive data must be normalised to eTh values. However, the majority of thorium normalisation and ratio maps were used in oil exploration, as reported by^10,44–46^ for gold mineralisation in altered areas.

The original method for locating hydrothermal sites in Crixias-Guarinos (Goiás, Brazil)^40^ was refined by^47^. Additionally, this method was applied in the studies by^48,49^. Unlike potassium and uranium, thorium has lower mobility. Hence, the optimal eU and K values are determined using thorium (eTh) as a lithological background monitor (Thorium normalisation)^50,51^. Based on radiometric data, hydrothermal alteration zones can be identified by the observed areas of K enrichment^43,45,52–54^. Shives et al.^55^ argue that low (eTh/K%) ratios are good indicators of hydrothermally altered zones; conversely, high (K%/eTh) ratios can be expected for hydrothermally altered zones. Ratio maps, such as (eU/eTh), (eU/K%), and (K%/eTh), created based on their close connection to mineralisation processes, are considered the best indicators for regions of uranium enrichment and potassium enrichment associated with hydrothermal alteration.

#### eU/eTh, eU/K ratio maps

**Figure 5:**
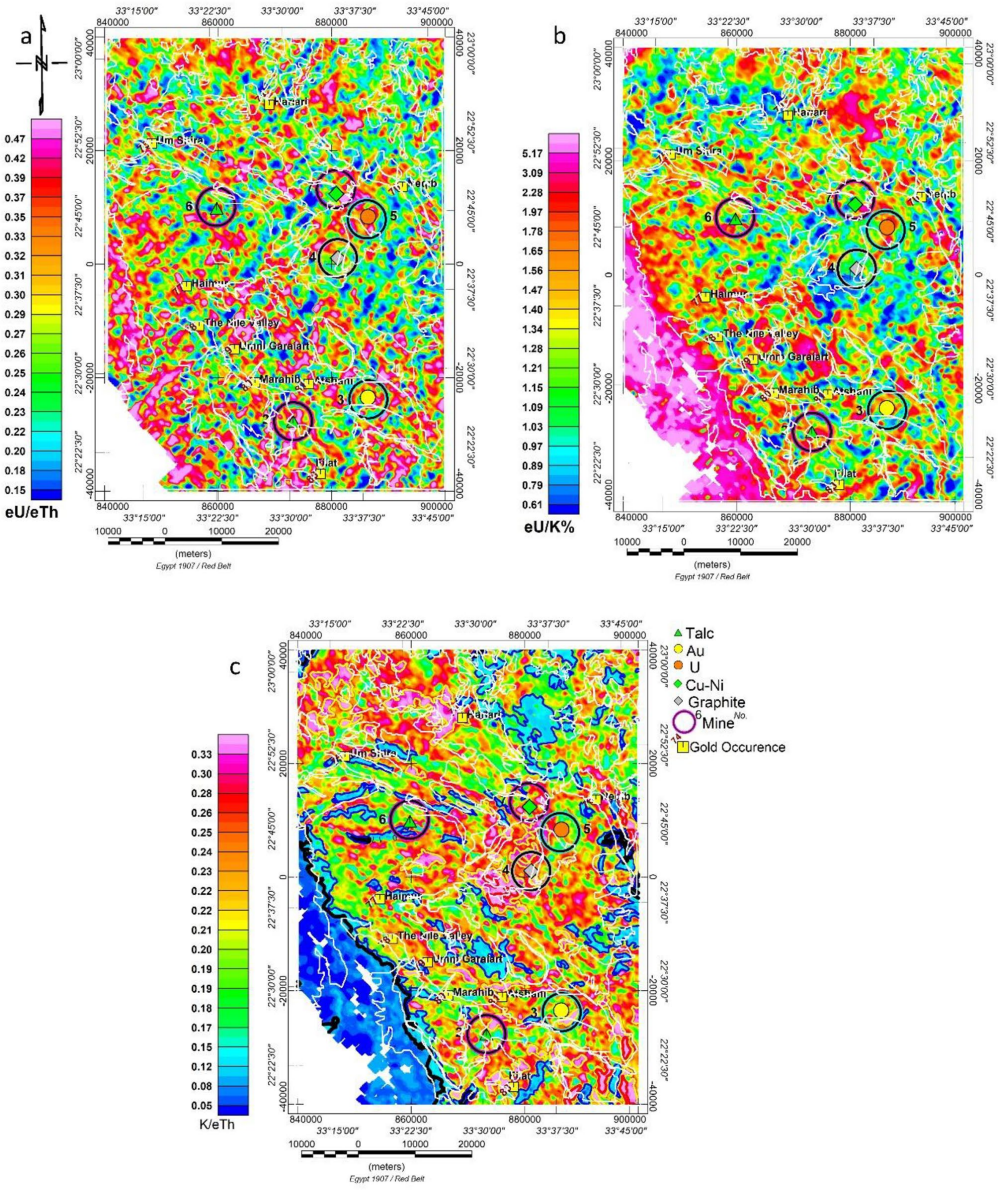
Ratio maps of the study area. (**a**) (eU/eTh), (**b**) (eU/K%), and (**c**) (K%/eTh). White lines represent the main geologic units, as shown in Fig. 3. Maps are produced using Oasis montage V 8.4 software^29^.

A favorable recognised trend of high ratio values is in the NE–SW direction, parallel to the drainage pattern and Wadis direction, as shown in Fig. 5a,b. However, high ratio values on eU/eTh and eU/K % ratio maps are unevenly distributed and spread throughout different geological units. The westernmost corner of the map shows an increase in the ratio values of eU/eTh and eU/K% on sedimentary rocks (> 5 ppm/% for eU/K). Because uranium is mobile and leaches, the high ratio in this region may be due to either a high noise-to-signal ratio or the leaching process.

#### The (K%/eTh) ratio map

Given that potassium has higher mobility than thorium and that the eTh/K% low ratio is a sign of regions of potassium enrichment associated with hydrothermal alteration, Gnojek and Prichystal^56^ used this ratio as an indicator for hydrothermal alteration. According to Hoover and Pierce^57^, the potassium-to-thorium ratio in most rocks is essentially stable, typically between 0.17 and 0.2 (K/eTh in%/ppm). The 0.1–0.17 ratio value characterises the sedimentary cover (at the western south corner) and the ophiolite serpentine, talc, and ankerite (osta) deposits. The K/eTh map, as given in Fig. 5c, has minimum and maximum values of 0.01 and 0.97, respectively. A few meta-volcanic and intermediate to Basic volcanic units (schist and tuffs chlorite-rich) (mvic) with a high K/eTh ratio of (0.4–0.5) are surrounded by ophiolite rock deposits (osta) and (ota) near the centre and south of the research region. Most recorded mineral deposits and gold occurrences are located in areas with a ratio value of (0.25–0.30) (%/ppm) units, which may suggest a moderate degree of alteration.

#### Potassium percent values deviation parameter (Kd%)

**Figure 6:**
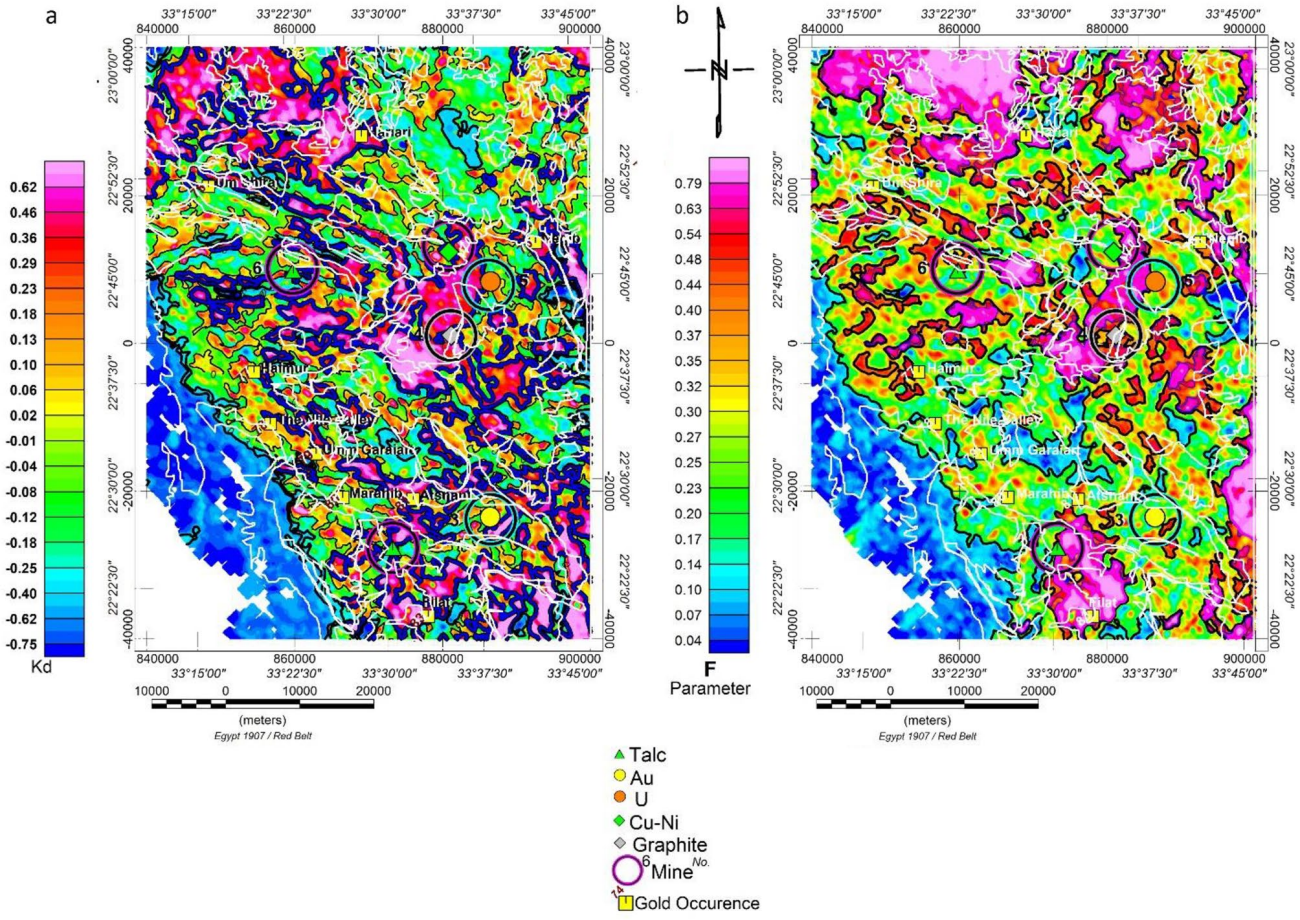
(**a**) Airborne gamma-ray spectrometric Kd% map, and (**b**) Airborne gamma-ray spectrometric map of F-parameters process. White lines represent the main geologic units, as shown in Fig. 3. The maps are produced using Oasis montage V 8.4 software^29^.

To map hydrothermal alteration haloes^40^, proposed a deviation from the ideal K values (Kd). The values for the nominal Kn were calculated using Eq. (2), where the statistics of the K and eTh grids yielded values of 1.18% and 5.6 ppm, respectively, for the K map average and eTh map average. As shown in Fig. 6a, the map was created by deviating from the nominal K values (Kd), which were thought to represent K enrichment values brought on by hydrothermal alteration processes. This may be expressed as:

1 $${\text{Kn}} = {\text{Th}}\;map*\left( {{\text{K}}\;map\;average} \right)/\left( {{\text{Th}}\;map\;average} \right)$$

2 $${\text{Kd}} = \left( {{\text{K}}{-}{\text{Kn}}} \right)/{\text{Kn}}\left( {3} \right)$$

**Figure 7:**
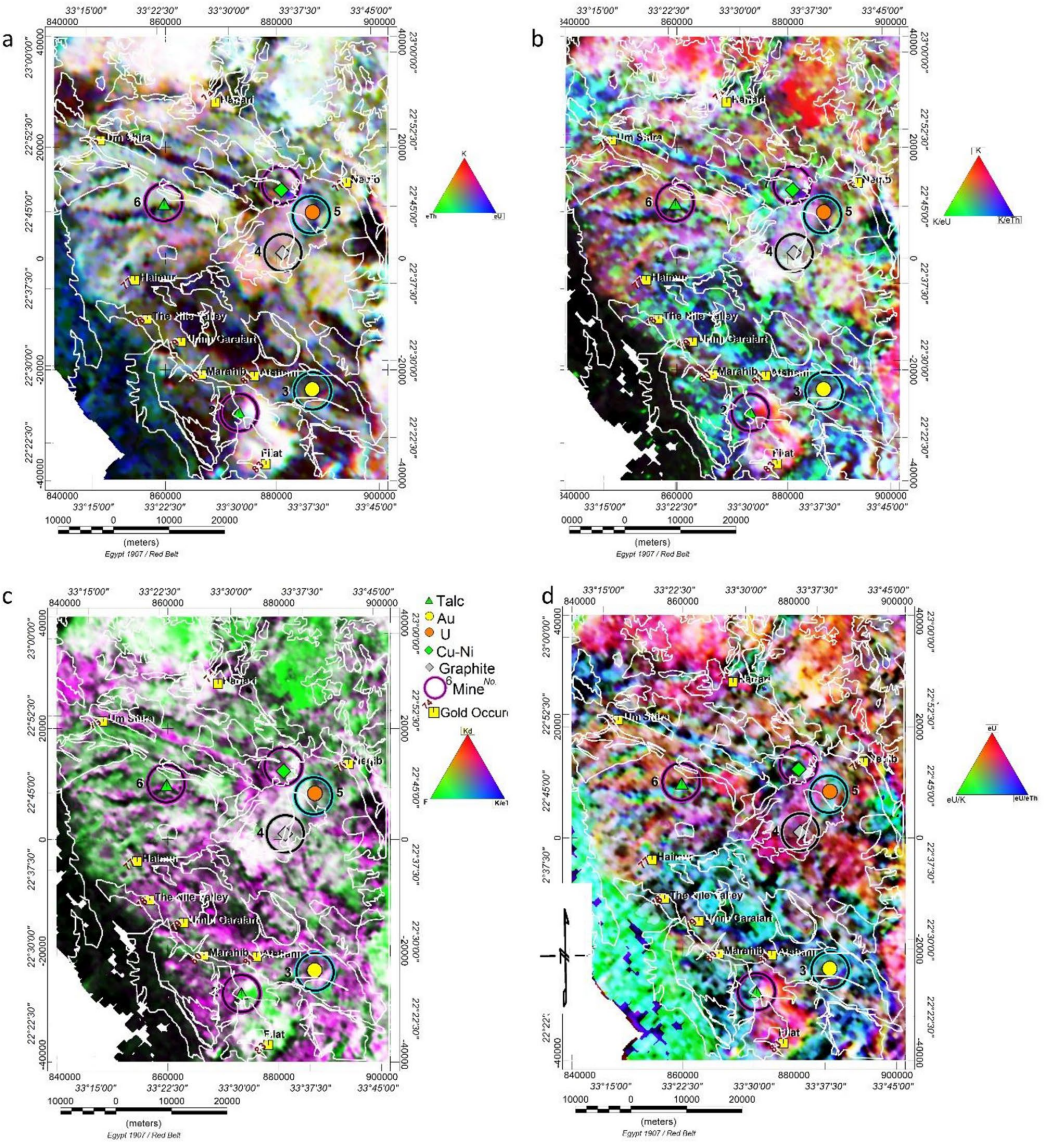
Airborne gamma-ray spectrometric ternary composite map of (**a**) (K, eTh and eU), (**b**) (K%, K%/eU, and K/eTh), (**c**) (KD, K/eTh, and (**d**) (eU, eU/K, and eU/eTh). The maps are produced using Oasis montage V 8.4 software^29^.

The Kd% map's minimum and maximum values are, respectively, −1.0 and 1.5. According to research, alteration zones with high Kd% values are thought to be connected to mineral deposits. The departure from nominal K values (Kd) (> 0.2) was thought to represent K enrichment values brought on by hydrothermal alteration processes, as illustrated in Fig. 7a.

To distinguish between the nonradioactive sedimentary cover and mafic and ultra-mafic intrusions (gbh, gbp) and the ophiolite rock group (osta), a contour value of (− 0.5 to − 0.3) per cent of Kd was found to be a good value. The 0.20 Kd per cent value contour was selected as a suitable value to indicate the delineation limit that passes through or close to the known mineralised sites and gold occurrences (Au, U, Cu–Ni, talc, and graphite), (as decided from comparison with published surface mineral occurrences from metallogenic map 1988), as shown in Fig. 6a. The majority of mineral deposits and gold occurrences are found in intermediate altered zones, or K-enriched sites, with a Kd% of (0.2).

### The F-parameter technique

The F-parameter technique proposed by Efmov^58^ is the ratio of the product of the K% and eU concentration to the eTh concentration. It was also applied because it involves two significant relationships: K richness correlated to the eTh/eU ratio or eU richness correlated to the eTh/K ratio. An F-parameter value of (1.2–1.3) indicates unaltered rocks, whereas a value of (2–5) indicates altered rocks^58^. The calculated F-parameter (as in Eq. 3) was gridded and presented as a colour grid image, as shown in Fig. 6b.

3 $${\text{F}} = {\text{K}}*{\text{eU}}/{\text{eTh}}$$

The F-parameter grid's computed minimum and maximum values in this study region were 0.01 and 2.41, respectively. Comparing F-parameter values derived by Efmov appears inappropriate for this region because F-parameter values greater than 2 (which indicate altered areas) are highly uncommon due to an alteration zone. Moderate F values > 1.2–1.3 are only present in spots where post-tectonic granitoids have been exposed and at a few other random locations. Therefore, the following alternative (F-parameter) boundaries were graphically selected:

The mafic, ultra-mafic, and ophiolite rock groups and sedimentary covered deposits are well-defined by the 0.15 F-parameter value contour. The sedimentary cover below this value is nonradioactive. Microgranite, monzogranite, and tonalite (qf, gm, and gt) rock types with substantial radioactivity are post-tectonic granitoids well-delineated by the 0.60 F parameter value contour. The 0.40 F-parameter value contour was chosen to travel along or very near the reported mineralised sites (Au, U, Cu–Ni, talc, and graphite), as shown in Fig. 6b.

Notably, two Talc sites, graphite and uranium occurrences, are situated in high or near-high F-parameter values (0.4 and 0.86, 0.4, 0.3, respectively). In contrast, Au and Cu–Ni occurrences are situated in an intermediate (0.19, 0.21) F-parameter value area (green to yellow colour). The F-parameter value of the gold occurrences was roughly 0.3.

### Ternary maps

Composite colour images combine data from various data sets into a single presentation. Such images make it easier to distinguish feature correlations in the various input file sets^59^. By modulating the red (R), green (G), and blue (B) colours of the display device in proportion to the concentration values of the three elements and performing visual spatial interpretation to distinct area colour degrees, a ternary colour composite image is produced. A 100% concentration of the radioelement is shown by the.

The colour is at each corner of the triangle. Different ratios of the three constituent elements are represented by the colors at each point inside the triangle.

Different ternary images of (RGB) were constructed as (K%–eTh–eU), (K%–K%/eU–K%/eTh), (eU–eU/K%–eU/eTh) and (Kd%–F–K/eTh) of relevant grids. Respectively, as shown in Fig. 7a–d, subtle variations in the elements (ratios) of the three colour bands to the radioelement or component distribution in the study area are revealed. Tertiary composite images could be considered a complex rock unit mapping method by recognising different radiometric element concentrations.

#### The (K%–eTh–eU) ternary composite image

**Figure 8:**
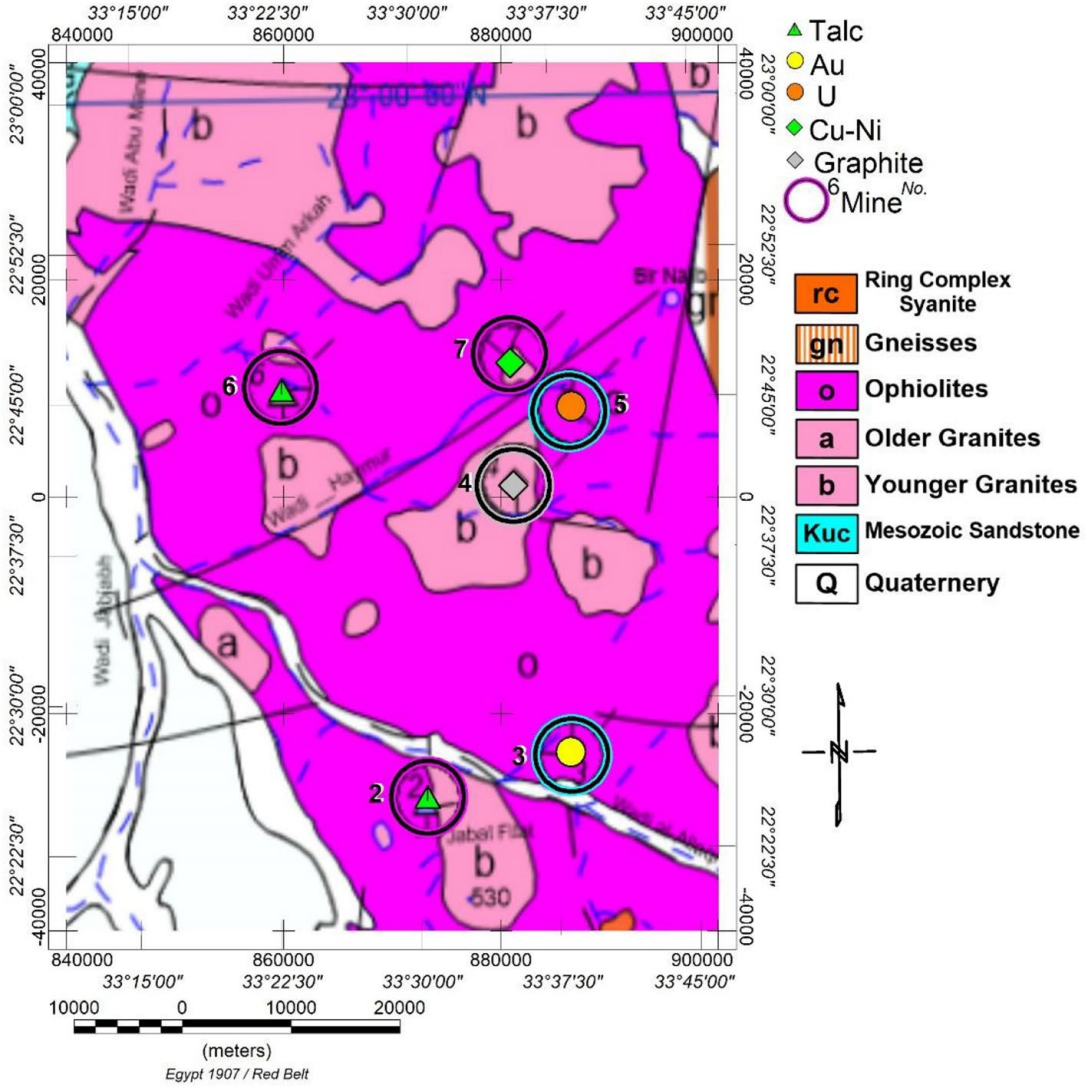
Metallogenic map of WA area, Eastern Desert, Egypt, after^23^. The map is compiled and modified with permission using Oasis montage V 8.4 software^29^.

As depicted in Fig. 8a, white and near-white areas are characterised by high concentrations of K%, eTh, and eU, accompanied by post-tectonic granitoid rocks (younger granites such as monzogranite, tonalite, and micro-granite). Dark (black) areas exhibit lower concentrations of the three elements. They are associated with wadi alluvium, intermediate volcanics, sub-volcanic rocks, ophiolite rocks (serpentine, talc, ankerite), as well as mafic and ultra-mafic intrusions (gabbro-dolerite).

The alternating green and blue mixture in the southwestern corner indicates Mesozoic sandstone, especially kaolinitic sandstone, characterised by very poor K content. A light pink and violet colour area is associated with some younger granite (tonalite) rock units due to low eTh and increased K% concentration.

The (K%–eTh–eU) ternary composite image is a valuable tool for geological mapping of rock units, providing insights into these formations' history and recent status. For instance, young granitoids display a reasonable K% content compared to old granitoids, which have undergone alteration, leaching, and mechanical and chemical weathering processes. The bluish or greenish colour indicates a decrease in K%, while reddish colours suggest areas of precipitation and fluid movements, particularly near wadies, appearing in bright or reddish colours. Greenish white is characteristic of meta-sedimentary rocks such as marble and schist, particularly those rich in sericite.

#### The K%–K%/eU–K%/eTh composite image

Figure 7b emphasises areas with a high K percentage, indicating altered regions. A white to the pale pink area is visible at the centre of the map, accompanied by tonalite (gt). The bright red area appears in the northeast corner, associated with younger granites partly covered by wadi alluvium. This red colour reflects a source percentage of K, with low K%/eU and K%/eTh ratios, indicating a leached source of K concentrations. As the K%/eTh ratio increases, the bright red colour transitions into pink, characterising granites in the northwest and some parts in the south of the same rock type.

Bright blue areas form a strip of 1.5 km in width and 31 km long, oriented NW–SE in the north and some rectangular areas in the southern region. All mineral occurrences are situated within these bright blue or green zones. The gold occurrence is located at the boundary between the white to bright green colour and the bright blue areas.

Dark (black) areas, characterised by low K%, eU, and eTh content, are indicative of wadi alluvium, intermediate volcanics, sub-volcanic rocks, ophiolite rocks (serpentine, talc, ankerite), as well as mafic and ultra-mafic intrusions (gabbro-dolerite). The Potassium Ternary Composite Image (Fig. 7b) of the study area is compared with the K%/eTh map (Fig. 5c), revealing hydrothermal alteration zones such as potassic and phyllic altered areas. These zones are closely associated with orogenic gold mineralisation and other mineral deposits.

The examination of results showing anomalously high Kd (0.2–1.5), high F-Parameter (0.4–1.2), abnormally high K/eTh (0.20–0.40), and bright spots on the Potassium Ternary Composite Image (Fig. 7b) suggests the presence of highly fractured and hydrothermally altered zones within the study area. These signatures highlight potassium enrichment halos associated with orogenic gold mineralisation in the study area.

#### The eU–eU/K%–eU/eTh composite image and Uranium anomalous areas

The relative uranium concentration concerning potassium and thorium is a crucial diagnostic factor for identifying potential uranium deposits^60^. Aerial gamma-ray spectroscopic survey data are primarily used to delineate potential uranium-rich areas' expected boundaries within various rock units^61^. The key parameters measured include the relative concentrations of uranium to thorium and uranium to potassium, in conjunction with direct uranium measurements.

In this context, the eU ternary composite image in Fig. 7d offers valuable visual information for identifying anomalous zones with enriched uranium concentrations. Additional criteria for delineating high eU areas involve the point's location with values greater than X + 2S for the same point, where (X) is the arithmetic mean and (S) is the standard deviation for the relevant eU, eU/eTh, and eU/K% maps. However, applying this criterion did not reveal anomalous areas within the study area, despite a recorded location of uranium resources with concentration values of (6.7 TC, 2.08 eU, 6.89 eTh, and 2.0 K% map units).

As depicted in Fig. 7d, the green-cyan colour (indicating a high eU/K% ratio and low eU concentration) shows a good correlation with sedimentary cover in the southwestern corner and ophiolite rocks in the middle east of the area, despite the low radioactivity of this region. There are no bright white areas, signifying high (eU, eU/eTh, and eU/K%), in the study area map, indicating the absence of anomalous regions for uranium ore. However, areas of red–purple colour characterise granitoid, with purple indicating the recorded uranium occurrence.

#### The Kd%-F(parameter)-K%/eTh composite ternary image

As indicated in Fig. 7c, the K composite ternary image reveals that white colour areas are associated with younger granites (micro-granite and monzogranite), occasionally transitioning to green.

The black (dark) colour areas on the map correspond to sedimentary deposits, including wadi alluvium, Mesozoic cross-bedded and kaolinitic sandstone in the southwestern corner of the study area, as well as volcanics, sub-volcanic rocks (schist and tuffs chlorite rich), and ophiolite rocks (serpentine, talc, ankerite) (osta). The purple colour predominantly covers the southern part of the map, forming a small spot in the northwest associated with a strip of NW–SE trend, 1.5 km wide and 35 km long, composed of volcanic-sub-volcanic rocks (schist and tuffs chlorite rich). The bright blue and red colours are absent, indicating a direct proportionality between Kd and the K%/eTh ratio.

The bright green colour signifies an increased percentage of the F factor with a very low percentage of Kd and K%/eTh ratio. This colour is distributed in the northern part of the map, accompanying post-tectonic younger granite masses (tonalite–monzogranite) and some wadi tributaries in the eastern south of the map. The bright green colour is also associated with some sheared meta-sedimentary rocks (schist sericite rich). Talc occurrences are situated at the purple–black–green boundary. At the same time, Cu–Ni, U, and graphite are found around the white-coloured area (Tonalite–granitoid), which transitions to green or purple. Gold occurrences are located in the purple area.

## Results

The metallogenic map was constructed at a scale of 1:100,000, the geological map at a resolution of 1:250,000, and the aero-radiometry maps at a scale of 1:50,000, which explains some anticipated differences between the two maps. While the area does contain an occurrence of uranium ore^24^, as illustrated in Fig. 8, there are no anomalous uranium ore sites that surpass the X + 2S criterion value for eU, eU/eTh, and eU/K simultaneously.

A favorable correlation exists between radiometric (eTh, eU, and K%) maps featuring extremely low to low values and the spatial positions of Cretaceous to Quaternary sedimentary deposits. Similarly, there is a link between low to moderate values and the geographical positions of Ophiolitic rocks, with high radiometric readings associated with post-tectonic granitoids.

Ternary maps of K, eTh, and eU offer a diverse representation of lithologic variations through colour distinctions. The identified radioelement zones exhibit a robust geographical correlation with mapped lithology (Fig. 3). Important lithological connections derived from the geological map are superimposed on this composite image map, and numerous locations within the research region closely align with the mapped units. This correlation implies that the resulting radioelement concentrations often reflect the underlying lithology.

Post-tectonic granitoids in the study area are consistently characterised by their pronounced radiometric response, clearly visible on radiometric maps. These plutons can be easily differentiated from adjacent rocks, which exhibit lower radioactivity. The contact between both types is readily apparent and traceable through dense contours. Notably, these post-tectonic granitoids exhibit a strong spatial correlation with zones of anomalously high K, eU, and eTh background concentration levels.

The zones exhibiting abnormally high K/eTh ratios, with values exceeding the typical range of 0.25–0.30%/ppm, are considered favorable for mineralisation and alteration. In the research region, K/eTh ratio values surpass the established range, reaching up to 0.45%/ppm. These enriched regions are scattered across the research area and are predominantly associated with post-tectonic granitoids and ophiolitic rocks interacting with other rock groups.

The F-parameter map values range from 0.03 to 1 and are categorised into three tiers. The first tier, with values ranging from 0.03 to 0.15, is associated with sedimentary rocks. The second tier, featuring values from 0.15 to 0.5 and concentrated in regions displaying green, yellow, and red colours, is connected with ophiolitic rocks. Gold and (northern) talc sites are distinctly linked, with F values ranging from 0.15 to 0.3. The third tier, with values from 0.6 to 0.9 and defined by violet-coloured granitoid rocks associated with post- and syn-tectonic granitoid rocks, includes most mineralisation locations, such as talc, U, Cu–Ni, and graphite. These are typically situated on the exterior edge of the (0.6–0.7) F contour.ss.

The "Kd%" map exhibits values ranging from − 0.75 to more than 0.80, with all gold and other mineral sites associated with 0.2–0.3 contour values bordering on the red colour. The map can be divided into three levels: starting with the lowest level, which has values ranging from − 0.75 to − 0.2 and is coloured blue, primarily located in the southwest and connected to sedimentary rocks. The second level, coloured green, features values ranging from − 0.2 to + 0.3 and is associated with ophiolitic rocks. The third level, with values ranging from 0.3 to 0.8, is connected with post-tectonic granitoids.

The K/eTh map indicates that all gold mines or locations and other minerals exhibit high or medium K/eTh ratio values. The northwestern, southeastern, and central regions of the research area show high values, while the southwestern corner, occupied by sedimentary cover, demonstrates low values. Comparing the K/eTh map to the KD% map reveals a complete resemblance (mirror image), with the same description applied, except that its values range from 0.05 to 0.4%/ppm.

**Figure 9:**
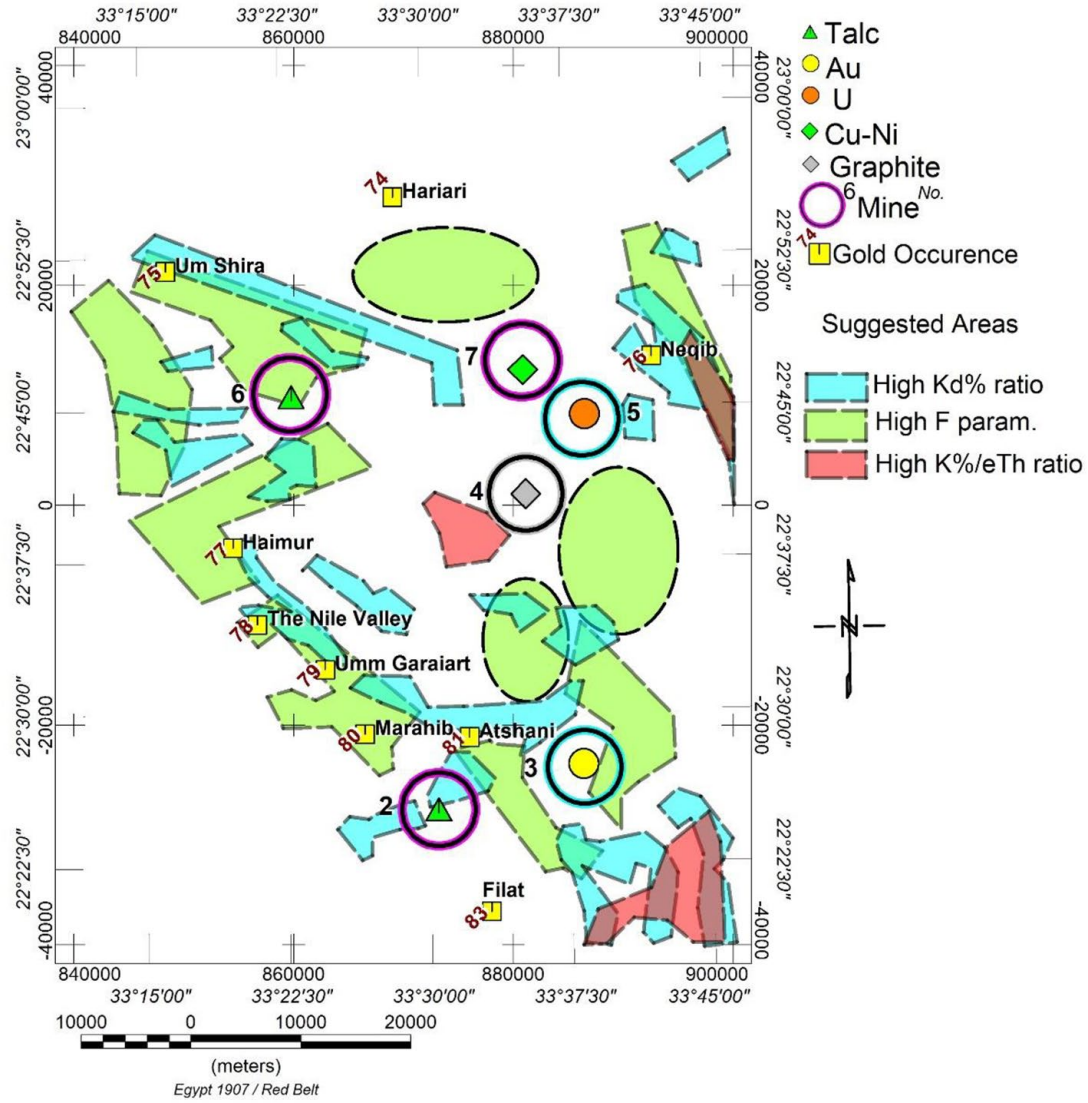
Suggested areas (High Kd, F param., and K/eTh).

The information derived from the three maps (K/eTh, KD%, and F Parameter) reveals anomalous hydrothermal locations in the examined region. It can be integrated to establish mineral favorability zones after excluding areas occupied by granitoids, which naturally exhibit high radiometric properties, zones with high Kd (blue colour), F parameter (green colour), and K/eTh (red colour) from their respective maps were identified. These selected and outlined zones are considered a priority as exploration targets for future geophysical and geochemical plans (Fig. 9). To simplify the map, some recorded mineral deposits located on the contact outside these granitoids in the outlined zones were not included.

Given that high K/eTh values suggest hydrothermally altered zones in the examined area, a ternary RGB map (KD, F-parameter, and K/eTh) was generated. It highlights hydrothermally altered regions where all three metrics strongly correlate, appearing white and indicating significant mineral favorability for Au deposits in the examined location. Purple-coloured patches represent hydrothermalised areas with a high association between KD (R) and K/eTh (B). The purple colour is linked to the Um Garayat gold mine, where both KD and K/eTh values are high, and the F value is low.s

### Comparison and validation of results

The findings of the current study align with those of^45,46^. In some gold mines, deficient levels of radiometric properties were recorded by El-Sadek.

The field geologic study undertaken in this research focused on examining 10 sites with gold occurrences and 5 sites featuring various types of mineralisation, including U, Cu–Ni, talc, and graphite of metallic ore and nonmetallic deposits. Most of these deposits exhibit low radiometric levels, falling within or near the boundary of high radiometric anomalies, except for the U types, which are characterised by high radiometric levels.

According to findings from^56^ and^58^, F-parameter values associated with alteration range from 1.2 to 1.3 for unaltered rocks and 2–5 or greater for altered areas, metallic ores, and nonmetallic deposits. The present study introduces new boundary values: 0.03–0.15 for nonaltered rocks and 0.3–6 or greater for altered areas.

As reported in^54^, the K/eTh ratio linked to hydrothermal alteration zones is approximately 0.3687. In the present study, this ratio varies slightly, ranging between 0.23 and 0.30 for the mineralisation sites under investigation.

## Conclusions

Gamma-ray spectrometry is commonly employed to augment regional geological mapping and aid in mineral prospecting, particularly for uranium. The data derived from gamma-ray spectrometry have traditionally played a crucial role in identifying regions of hydrothermal alteration and establishing their connection to the mineralisation processes of various metals, including base metals, gold, and silver, across diverse geological environments. It is important to note that the relationship is indirect; radiometric data is utilised to identify areas of hydrothermal alteration, which may or may not be consistently associated with mineral deposits.

This work utilises airborne spectrometric data processing to generate radiometric maps of Um Garayat's radioactivity. Various ratio maps, such as the K/eTh ratio map and Ternary Composite Images (Potassium, F-Parameter, and Kd) (potassium anomalies), were employed to identify hydrothermal alteration zones closely associated with gold mineralisation. The Potassium Ternary Composite Image revealed abnormally high Kd values (0.3–1.7) and F-Parameter values (0.6–3.5), as well as elevated K%/eTh ratios (0.33–0.77) and bright patches, indicating the most likely hydrothermal zones within the studied area. Radiometric data boundaries associated with hydrothermal alteration are considered favorable areas for further exploration. These distinctive features, identifiable by K enrichment halos, are linked to gold and mineralisation sites in the research region.

The processing methods supporting these findings validate the correlation between known gold deposits, metallic ores, nonmetallic deposit occurrences in the studied area, and hydrothermal K. The connection between K enrichment, hydrothermalised areas, and mineralisation events can be elucidated by the ore-deposit model developed for Um Garayat and its environs.

It was explained in this way that hydrothermalised zones were discovered by high values of the “F-parameter,” "Kd%," and the K%/eTh ratio. A ternary "RGB map" may readily integrate data for mineral favorability. This data integration (R = KD%, G = F parameter, and B = K/eTh) confirmed extremely favourable zones centred on white–purple contact regions. We may propose a new exploratory target, as shown in Fig. 9, characterised by high F, Kd, and K/eTh values. The known gold mine is associated with deficient aero-radiospectrometric levels on all four maps.

The gamma-ray spectrometry method is widely utilised in many sectors, including uranium exploration, geological mapping, mineral discovery, and soil mapping. The current work demonstrates hydrothermalised zones regarding K abundance and how it relates to nonmetallic deposits, metallic ores, and gold.

## Data Availability

The datasets used and/or analyzed during the current study are available from the corresponding author upon reasonable request.
